# On Inflammation of the Bowels

**Published:** 1806-06

**Authors:** 


### *To the Editors of the Medical and Phyſical Journal.*

GENTLEMEN,

IF the following, what I would call occasional report of diseases, comes into your plan, and so far meets with your approbation as to appear in your Journal, I shall be encouraged to go on; I shall not be discouraged, I hope, if it is not thought worthy; or shall I, in consequence, be inclined to quarrel with your publication, a reader of which I hope I always shall be, so long as I receive pleasure and improvement therefrom.

Two cases of inflamed bowels; one case of puerperal fever; two of inflammatory rheumatism; two of apoplexy; and several inflamed tonsils, not in every one amounting to suppuration, have been the most prominent cases that have passed in my practice lately. The cases of inflamed bowels bowels were in subjects quite opposite to each other in constitution; the one being a delicate young woman, the other a labouring man, above forty, stout and robust. In neither case was there any distinct rigor before the attack of pain; the former had had a cold upon her some time, from which she thought herself nearly recovered; after walking out on a cold day, she was seized in the evening with violent pain in her bowels, attended with severe vomiting and purging; it was on account of these evacuations that I was first called to see her the next morning. By the purging I was deceived, for I immediately concluded that it was a common bowel complaint, and ordered the chalk mixture with cinnamon; pretty early in the afternoon I saw her again; the purging I found had ceased, but the vomiting and pain were still going on; these continuing, roused my attention, and I now had to regret the loss of some hours, in pursuing a useless, if not pernicious remedy. The pulse was very quick and hard, thirst extreme, urine very high coloured, belly very much swelled, and tender, and sounding when gently struck upon; breathing laborious, great restlessness, occasional hiccup, and sunk countenance; these, with the pain and vomiting before mentioned, make a formidable and truly unequivocal set of symptoms. Repeated bleeding and purgatives; but perceiving that these were immediately rejected, opium and purgatives alternately, glysters, a blister to the abdomen, and, in the end, warm bathing, succeeded in subduing the disease. In the man cold also seemed to be the exciting cause of the complaint; his sufferings were miserable to behold; his belly was swelled and sore, but in point of feeling very unlike the former case; no elasticity, no hollow sounding, which was attributed to the strong and unyielding action of the abdominal muscles; this unaccommodating state of muscular fibre, was thought also to contribute to his pain, which appeared indeed almost intolerable; pulse quick, but soft; vomiting almost incessant, thirst great, urine not remarkably changed; these were his symptoms. Large bleedings; after the second bleeding the warm bath as long as he could bear it, which was a little more than an hour, and a grain of opium swallowed when going into the bath, abated his pain and sickness much; the disease, however, did not immediately yield, but another bleeding, with opiates and purgatives alternately, glisters, a large blister, and a second bathing, brought about a happy change, and rescued him from a situation that appeared almost desperate. Opium in both cases had the best effect, it gave immediate comfort to the patients, and prepared the stomach for the reception and better retention of purgative medicines.

If the puerperal fever is characterized by a rigour some days after delivery, succeeded with a violent pain in the abdomen, a hard pulse at 120 or upwards, thirst, headach, suppressed or diminished lochia; secretion of milk checked, or never appearing, generally swelled belly, or circumscribed very tender tumour; then have I seen many instances of that disease, of which I do not recollect an unfavourable termination since I have followed the plan recommended by Dr. Gordon. My practice before that time, I have pain in the remembrance, was not so fortunate; which I now attribute in part to my own then inexperience, and partly to the opposite views given of the disease by different authors; which, by making the steps of a young man fearful and indecisive, in a disease of such hasty termination, produce much evil.

A PRACTITIONER in *Derbyshire.*

*May* 5, 1806.

